# Supplementary material for: Magnetic and mechanical hardening of nano-lamellar magnets using thermo-magnetic fields
Source: Nat Commun. 2025 Mar 11;16:2423. doi: 10.1038/s41467-025-57571-6 (PMC11897201; doi:10.1038/s41467-025-57571-6)
Supplement: Supplementary file 1 — Supplementary Information [file 41467_2025_57571_MOESM1_ESM.pdf]

Supplementary Information

Magnetic and mechanical hardening of nano-lamellar magnets using thermo-magnetic fields

Liuliu Han[1*], Jin Wang[2], Nicolas J. Peter[2], Fernando Maccari[3], András Kovács[4], Ruth Schwaiger[2], Oliver Gutfleisch[3], Dierk Raabe[1]

[1]*Max Planck Institute for Sustainable Materials, Max-Planck-Straße 1, 40237 Düsseldorf, Germany*
[2]*Institute of Energy Materials and Devices, Forschungszentrum Jülich, 52425 Jülich, Germany*
[3]*Institute of Materials Science, Technical University of Darmstadt, 64287 Darmstadt, Germany*
[4]*Ernst Ruska-Centre for Microscopy and Spectroscopy with Electrons, Forschungszentrum Jülich, 52425 Jülich, Germany*

Correspondence to Dr. Liuliu HAN l.han@mpie.de

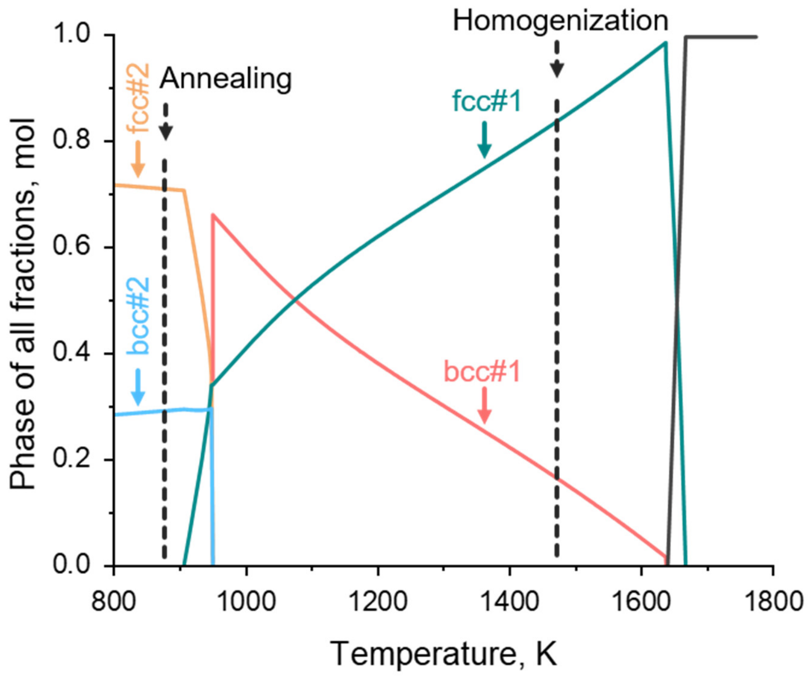

Supplementary Figure 1. Temperature dependence of the phase fraction evolution of the current Co-Fe-Ni-Al MCA.

For selection of the isothermal heat treatment temperatures, we used the thermodynamics software Thermo-Calc equipped with the High Entropy Alloy database (version 4.2). For precise prediction, the bulk chemical composition measured by wet-chemical analysis, i.e., $Co_{28.6}Ni_{28.8}Fe_{30.6}Al_{12.0}$ (at.%), was used to perform the simulation. The calculated equilibrium volume fraction and composition of the fcc#1 and bcc#1 phases at 1473 K is 14.9%, $Co_{24.1}Fe_{23.1}Ni_{29.3}Al_{23.5}$ and 85.1%, $Co_{29.5}Fe_{32.1}Ni_{28.6}Al_{9.8}$ (at.%).

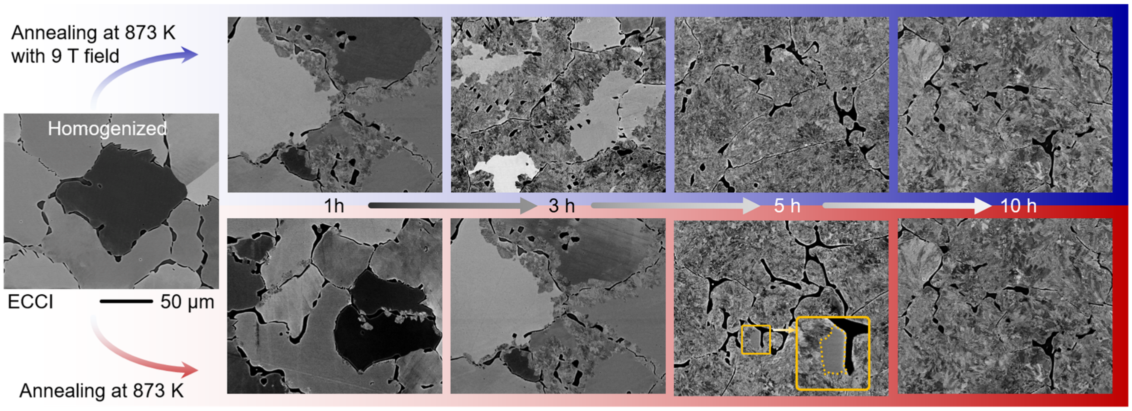

Supplementary Figure 2. **Evolution of eutectoid decomposition of the Co-Fe-Ni-Al MCA.**

Ex-situ ECCI experiment showing eutectoid decomposition during isothermal heat treatment under a magnetic field (top images) and without a magnetic field (bottom images).

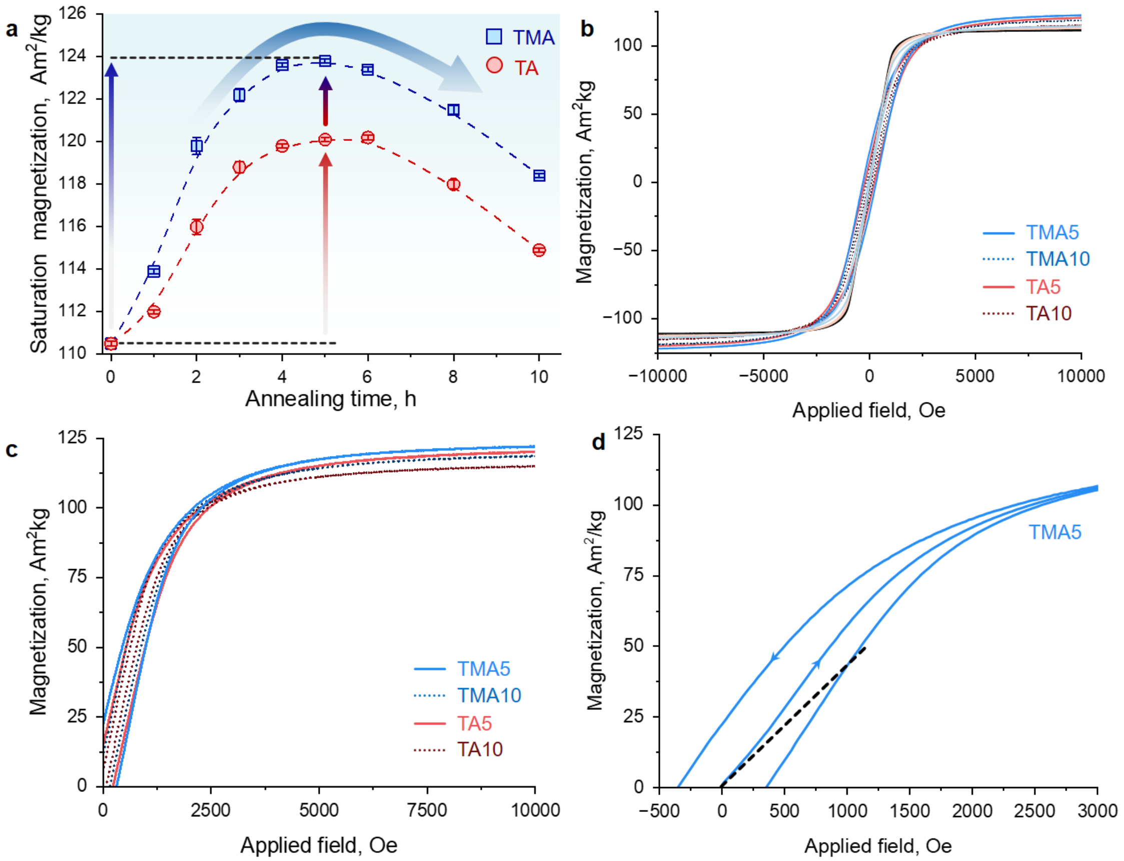

Supplementary Figure 3. Magnetic hysteresis loop analysis of the current MCAs.

a Average saturation magnetization (M_s) values versus annealing time during the TMA and TA treatments. **b** Room-temperature hysteresis loop of the current MCAs. **c** Enlarged view approaching the magnetization from the first quadrant. **d** Enlarged view of the hysteresis loop showing that the magnetization process of TMA5 is governed by pinning.

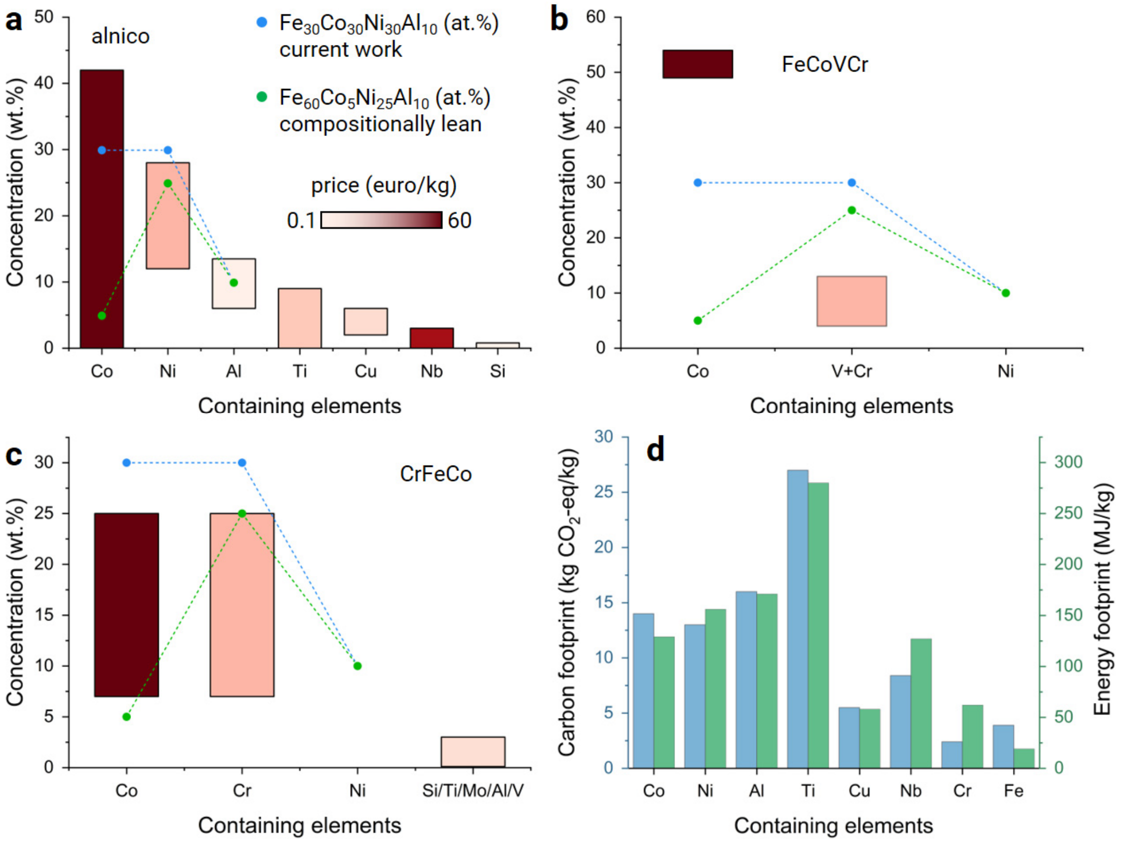

Supplementary Figure 4. Sustainable considerations including alloy cost, carbon, and energy footprint of the current MCAs compared to the commercial RE-free magnets.

Comparison of concentration and price of the containing critical elements of **a** commercial hard magnetic Alnico alloy, **b** semihard FeCoVCr and **c** CrFeCo alloys and the current FeCoNiAl and compositional lean variant FeCoNiAl. **d** carbon footprint and energy footprint of the critical elements[64] of commercial RE-free semihard and hard magnets.

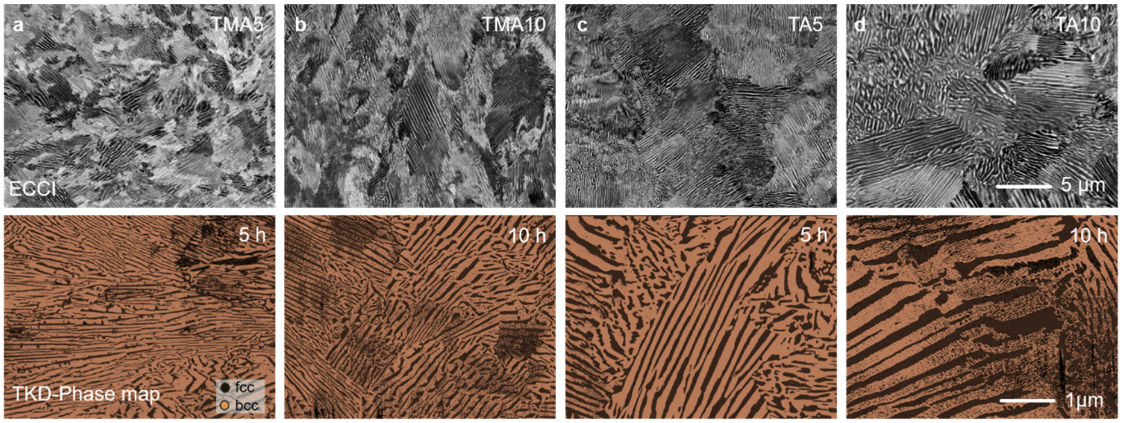

Supplementary Figure 5. ECCI and TKD characterization

ECCI and TKD analysis of the lamellar structure in **a** TMA5; **b** TMA10; **c** TA5, and **d** TA10.

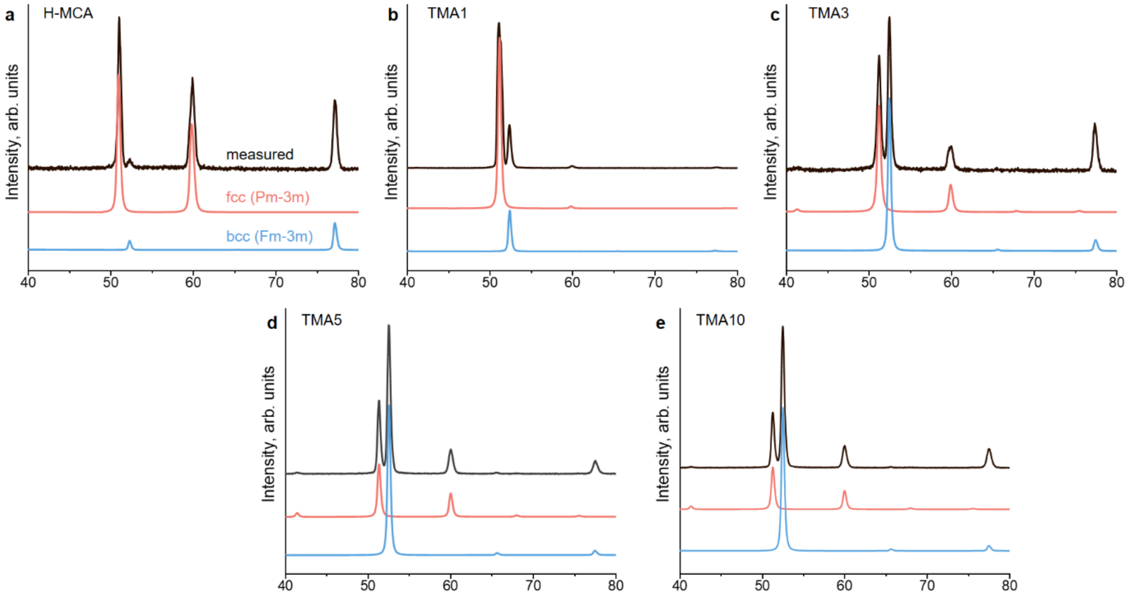

Supplementary Figure 6. X-ray diffraction analysis.

Measured and simulated XRD patterns showing the phase structure and texture of the investigated Co-Fe-Ni-Al multicomponent magnets of different thermo-magnetic annealing times. **a** H-MCA. **b** TMA1. **c** TMA3. **d** TMA5. **e** TMA10.

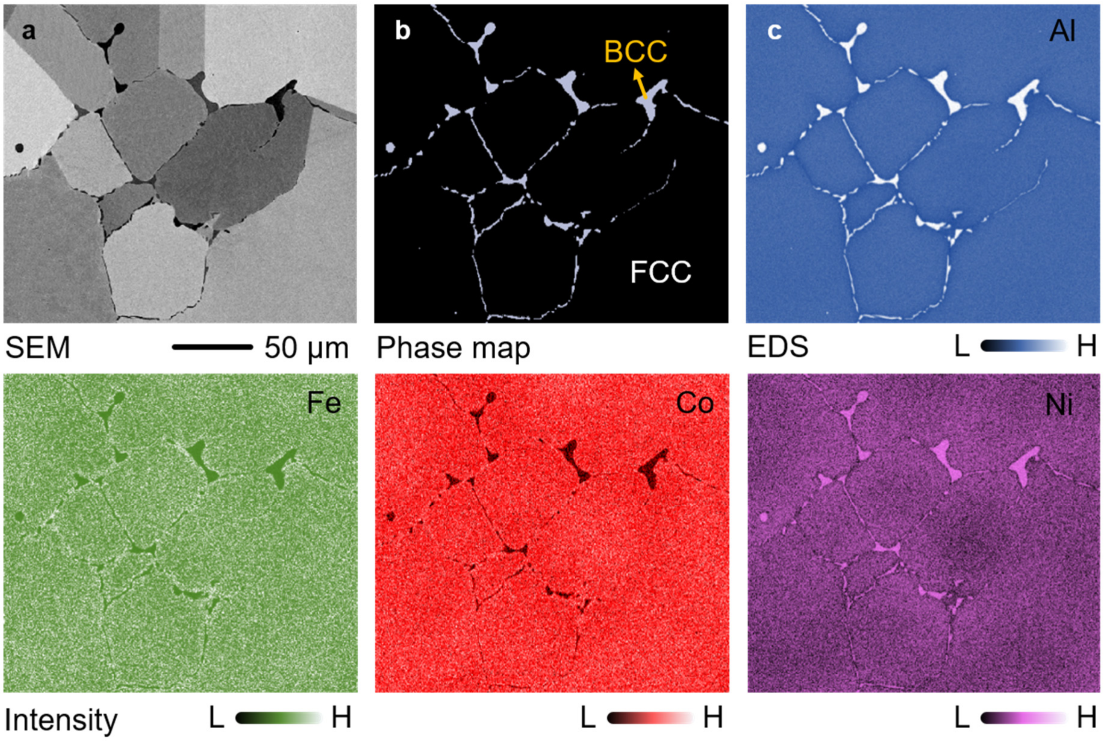

Supplementary Figure 7. Electron probe microanalysis of the H-MCA.

a Typical SEM micrograph showing the phase distribution in the H-MCA. **b** Corresponding phase map. **c** Elemental mappings. The average composition of the fcc and bcc phases is $Co_{29.2\pm0.4}Fe_{31.5\pm0.4}Ni_{28.1\pm0.5}Al_{11.2\pm0.4}$ and $Co_{22.3\pm0.3}Fe_{19.8\pm0.2}Ni_{31.4\pm0.3}Al_{26.5\pm0.1}$, at.%, respectively.

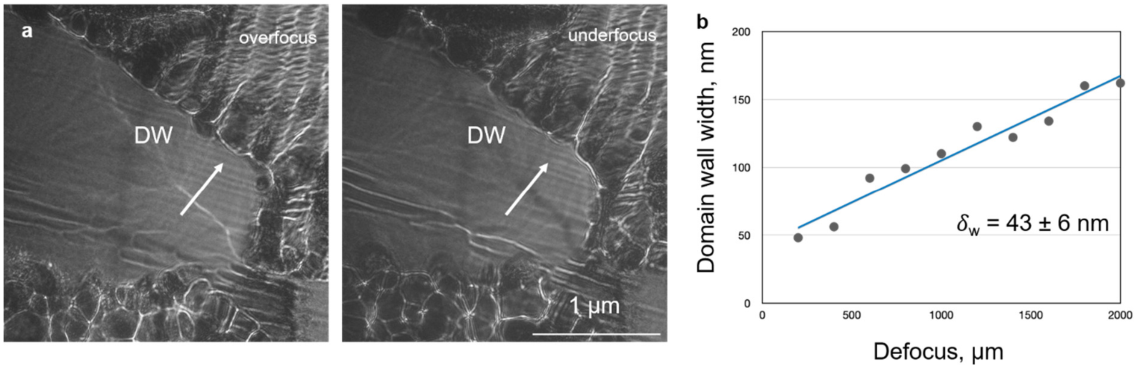

Supplementary Figure 8. LTEM analysis of the TMA5.

a Overfocus and underfocus Fresnel image of the domain pattern. The integrated intensity of the divergent wall contrast (marked by white arrows) was measured at different defocus values. **b** The full width of the half minimum is plotted as a function of the defocus. The extrapolation of the linear fit to zero gives a domain wall width of 43 nm.

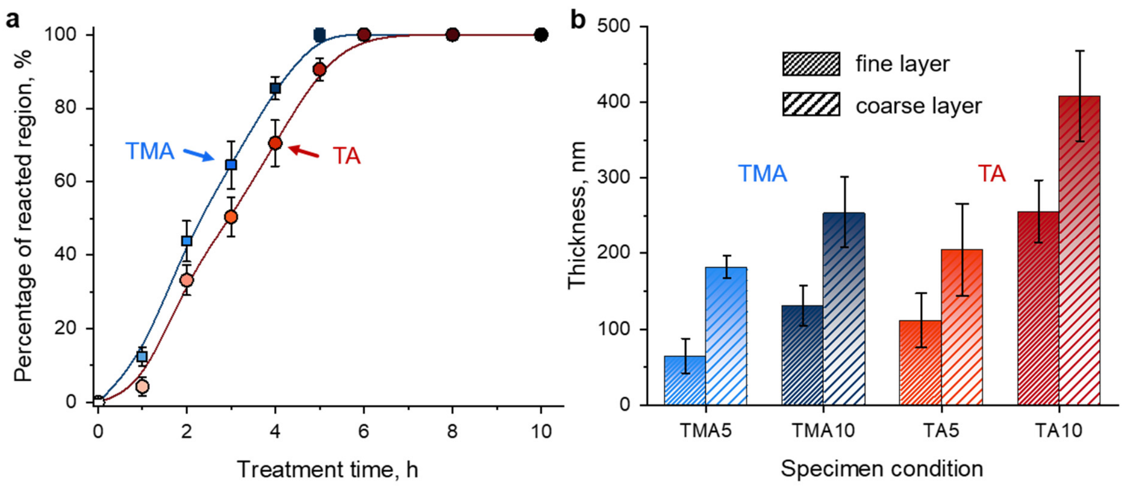

Supplementary Figure 9. Statistical analysis of the lamellar phases during different annealing processes.

a Evolution of the volume fraction of the eutectoid decomposition region during annealing. **b** Average thickness of the fine and coarse layers.

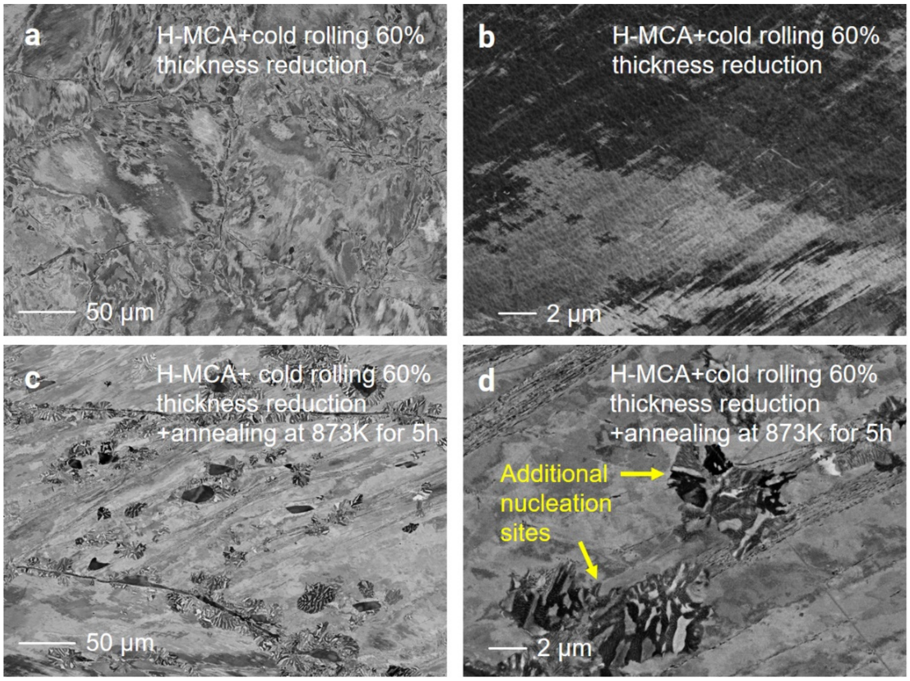

Supplementary Figure 10. SEM analysis of the current MCAs after different thermo-mechanical treatments.

a, b Typical ECCI images of the MCA after cold rolling the H-MCA for 60% thickness reduction. **c, d** Typical BSE images of the MCA after cold rolling the H-MCA for 60% thickness reduction followed by annealing at 873 K for 5 h.

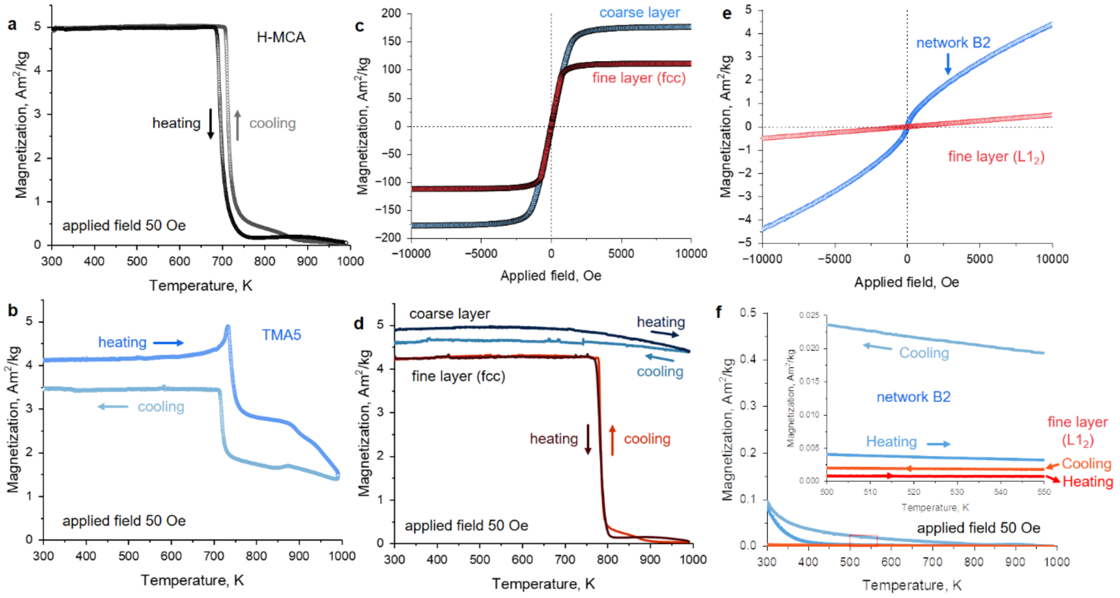

Supplementary Figure 11. Magnetic performance.

a In-situ magnetization-temperature measurement of H-MCA. **b** In-situ magnetization-temperature measurement of TMA5. **c** Room-temperature hysteresis loop of the bulk ingots with nominal compositions identical to those of the fine and coarse layers determined by APT and **d** corresponding in situ magnetization-temperature curves. Both the fine and coarse layers are ferromagnetic. The fine layer has a T_c of 774 K, while the coarse layer has a T_c >990 K. **e** Room-temperature hysteresis loop of the bulk ingots with nominal compositions identical to those of the network bcc and $L1_2$ phases determined by APT and **f** Corresponding in-situ magnetization-temperature curves. The $L1_2$ phase is paramagnetic, and the network B2 phase shows weak ferromagnetic behaviour.

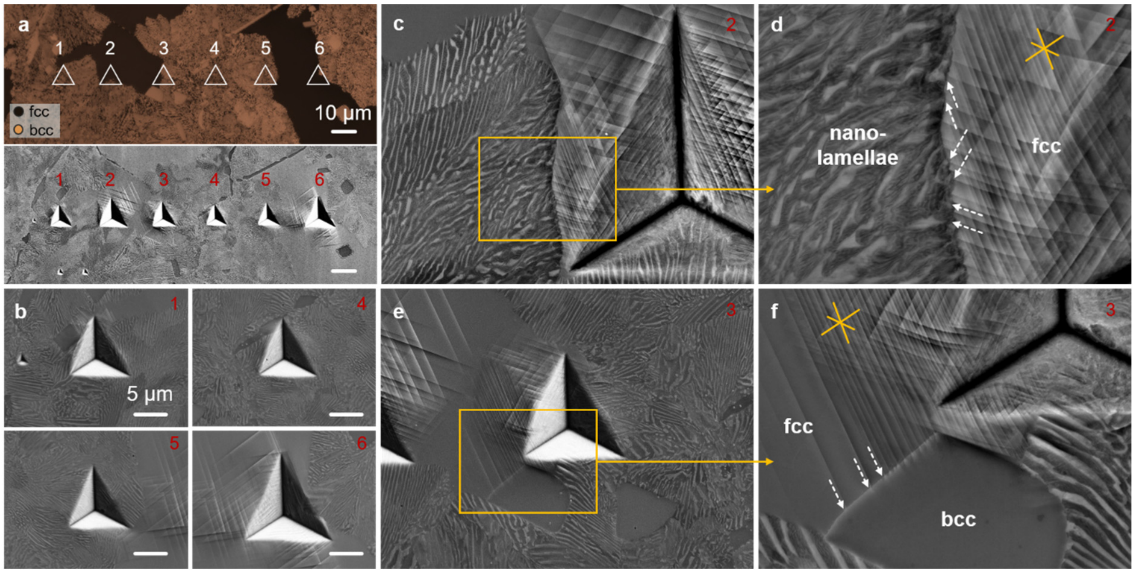

Supplementary Figure 12. Deformation and slip trace analysis after the nanoindentation measurements.

a EBSD phase map identifying two phases before (top) and an ECC micrograph after the indentation test (bottom), showing the location of the indentations and the surface morphology. **b** ECC image showing the difference in the residual area of the Berkovich indents located in different phases. The indent (#6) located mainly in the fcc phase had a larger residual area than the rest of the indents in the eutectoid lamellar region (#1, #4 and #5). **c** SEM micrograph showing an indent (#2) located in the fcc phase with its left side close to the eutectoid lamellar region. **d** Magnification identical to that of the solid frame in (**c**), showing that the activated $<110>_{fcc}$ slip lines were hindered at the eutectoid lamellar/fcc boundaries. **e** SEM micrograph showing another indent (#3) located in the eutectoid lamellar region with its left side close to the fcc and bcc phases. **f** The corresponding magnification identical to the solid frame in (**e**) showing that the activated $<110>_{fcc}$ slip steps were hindered at the bcc/fcc boundaries.

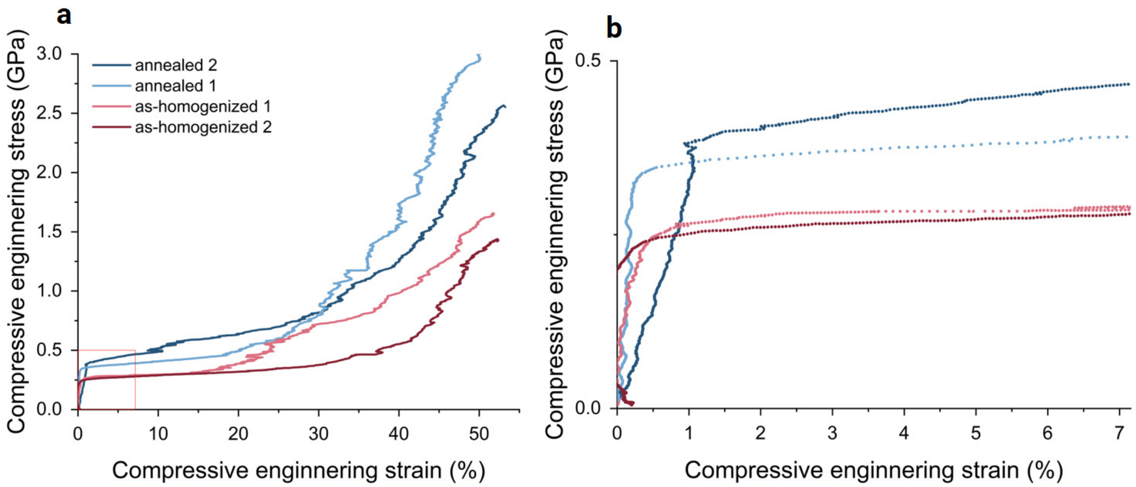

Supplementary Figure 13. Bulk mechanical performance of the Co-Fe-Ni-Al alloy with different microstructure features.

a Compressive engineering stress-strain curve of the as-homogenized (H-MCA) and thermal annealed (TA5h) alloys. **b** Enlarged view identical to the red frame in (**a**) showing the enhanced yield strength of the TA5h alloy containing eutectoid lamellae as compared to the H-MCA.

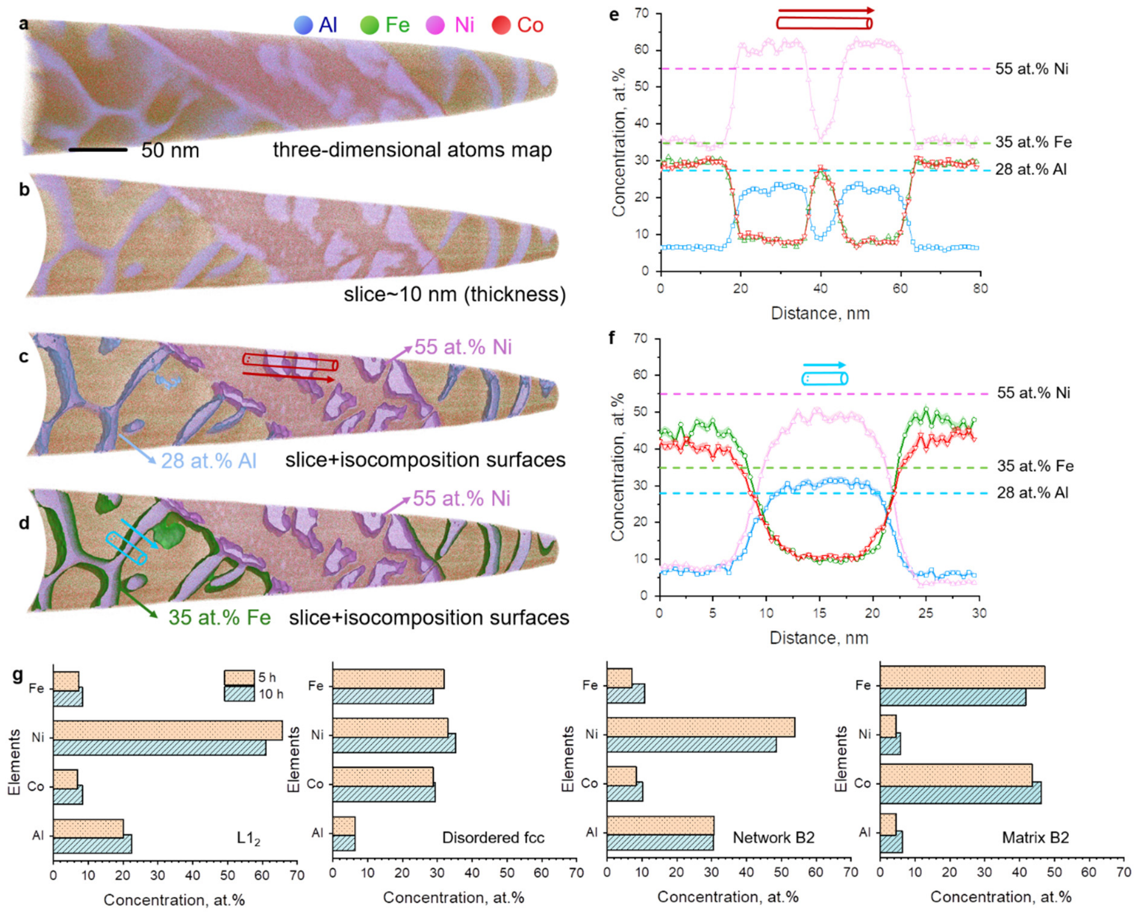

Supplementary Figure 14. APT analysis of the TMA10.

a Reconstructed 3D APT map containing all the elements from the eutectoid decomposition region. **b** 10 nm-thick cross-sectional slice from the 3D APT map. **c** The interface of the cross-sectional slice is highlighted with 55 at.% Fe+28 at.% Al. **d** The interface of the cross-sectional slice is highlighted with 55 at.% Fe+35 at.% Fe. **e** 1D compositional profiles of the red cylinder region along the red arrow marked in (**c**). **f** 1D compositional profiles of the blue cylinder region along the red arrow marked in (**d**). **g** Average composition of the phases contained in TA10 compared to those in TMA5.
